# Melamine Faced Panels Defect Classification beyond the Visible Spectrum

**DOI:** 10.3390/s18113644

**Published:** 2018-10-27

**Authors:** Cristhian A. Aguilera, Cristhian Aguilera, Angel D. Sappa

**Affiliations:** 1 Universidad Tecnológica de Chile INACAP, Av. Vitacura 10.151, Vitacura 7650033, Santiago, Chile; c_aguilerac@inacap.cl; 2 University of Bío-Bío, DIEE, Concepción 4051381, Concepción, Chile; 3 Computer Vision Center, Edifici O, Campus UAB, Bellaterra, 08193 Barcelona, Spain; asappa@cvc.uab.es; 4 Escuela Superior Politécnica del Litoral, ESPOL, Facultad de Ingeniería en Electricidad y Computación, CIDIS, Campus Gustavo Galindo, Km 30.5 vía Perimetral, Guayaquil 09-01-5863, Ecuador

**Keywords:** infrared, industrial application, machine learning

## Abstract

In this work, we explore the use of images from different spectral bands to classify defects in melamine faced panels, which could appear through the production process. Through experimental evaluation, we evaluate the use of images from the visible (VS), near-infrared (NIR), and long wavelength infrared (LWIR), to classify the defects using a feature descriptor learning approach together with a support vector machine classifier. Two descriptors were evaluated, Extended Local Binary Patterns (E-LBP) and SURF using a Bag of Words (BoW) representation. The evaluation was carried on with an image set obtained during this work, which contained five different defect categories that currently occurs in the industry. Results show that using images from beyond the visual spectrum helps to improve classification performance in contrast with a single visible spectrum solution.

## 1. Introduction

Companies face the great challenge of being competitive worldwide, which is expressed in many cases in the creation of new and better products, and in the improvement and innovation in their production processes. A key factor in the production chain is undoubtedly the quality control of their products. In this sense, it is increasingly necessary to incorporate new and better inspection processes of the final quality of their products.

In the wood industry, the production of wood-based panels has had a sustained growth in recent years. Different studies show that this market is very dynamic and generates sales for significant amounts of revenues every year. For example, the market size of wood-based panels is expected to reach USD 174.55 billion by 2025, a 7% of compound annual growth rate [1]. Products such as particle boards, oriented chipboards and plywood boards, among others, provide a wide variety of possibilities that are used by the construction industry and furniture.

The wood industry has incorporated methods of quality control of its products, adopting a series of technologies that facilitate both the best use of the material and the quality inspection of its products. This last point has a significant economic impact, both from the point of view of the best use of the raw material and of the final customers. Initially, these inspections were carried out by operators, but nowadays, with the advent of better technologies, this process is being replaced by specialized machines with a high degree of performance.

The classification of surface defects is one of the most important tasks in the quality control stage of panels with melamine surfaces; the main subject of study in this work. In particular, the classification of defects is of great importance, since on the one hand, it allows generating statistics that help to correct the processes that cause the defects and, on the other hand, they allow classifying the quality of the panels depending on the types of detected defects. Currently, there are numerous works that point to the automated classification of defects. However, aspects such as reflections on shiny surfaces, surfaces with low contrast, complex color relationships, small defects, complex designs, among others, make these automated solutions have poor performance when inspecting this type of surfaces. This paper presents an exploratory study of the use of images beyond the visible spectrum for the classification of defects in melamine based boards, providing useful insights regarding the real applications of the technology.

Numerous applications in the field of classification of wood surface defects have been proposed. Gu et al. [2] proposed a classification of different types of knots on wooden surfaces making use of the intensity and size features of these defects and applying a Support Vector Machines (SVM) classifier [3]. The work proposed by Mahram et al. [4] classifies different types of knots, using SVM, K-Nearest Neighbor (KNN) [5] and texture descriptors for feature extraction. Another contribution is presented by Yuce et al. [6], where different types of defects in wood veneers are classified using neural networks. In YongHua and Jin-Cong [7], a method of segmentation and classification of knots on wood surfaces is presented, based on Gray-Level Co-occurrence Matrix (GLCM) [8] and texture parameters of Tamura [9]. A particularly interesting work is presented by Hittawe et al. [10], which propose a method of detection and classification of knots and cracks in wood based on color images, using Local Binary Patterns (LBP) [11] and Speeded-Up Robust Features (SURF) [12] for feature extraction and SVM as a classifier. All the aforementioned works present methods applied to common wood surface defects, such as knots, cracks, stains, holes, etc. (e.g., [13,14]), without considering critical aspects in real inspection systems such as surfaces with low contrast, shiny surfaces, among others. Additionally, all the methods presented above are based on the usage of visible spectrum images, while our work consider the usage of cameras from different spectral bands to tackle the difficulties previously mentioned.

In the case of boards with melamine surfaces, Santiago et al. [15] shows an application for detection and classification of defects in real time on medium density fiberboard (MDF) boards with different texture designs, however, prior to the analysis the texture type must be set manually for each sample, besides being very sensitive to the type of lighting and reflections on the analyzed surface.

In the current paper an exploratory study for classifying defects in wooden boards with melamine surfaces using multispectral information is presented. The information is obtained from different cameras: visible (VS), near-infrared (NIR) and long wavelength infrared (LWIR), also referred to as thermal infrared. Especially, infrared cameras are capable of extract information that is not provided by the visible spectrum, such as relief areas or dents, cracks, moisture variances or different materials, among others. For example, Berglind and Dillenz [16] used thermography to detect glue problems in laminated wood; thermography is also used for the detection of defects in wood [17] and for detecting defects in wood-based products [18], such as laminated floors of high-density fiberboards (HDF).

Through experimental evaluation, we evaluate the performance of each spectral band, using a feature descriptor learning approach together with a support vector machine classifier. Two descriptors were evaluated, Extended Local Binary Patterns (E-LBP) [19] and SURF [12] using a Bag of Words (BoW) [20] representation. Melamine faced panels samples, that contained five different defect categories, were provided by a wood product company.

As a summary, the main contributions of this work are:

- We explore the use of images from different spectral bands to classify defects in melamine wood-based panels. Demonstrating, through experimental evaluation, the value of images beyond the visible spectrum in the classification of melamine faced panels.
- We show that the classification performance of melamine faced panels defects increases when different spectral bands are combined.

## 2. Problem Description

In essence, a melamine board is a particle board coated with a decorative sheet impregnated with melamine resins. The particle board consists of small fragments of pine, usually called pine shavings, crushed and selected to later mix with a special adhesive, generally made from water, resin, and chemical hardeners. After the mix is ready, the particle-board and the sheet are fused using a heating system.

**Figure 1:**
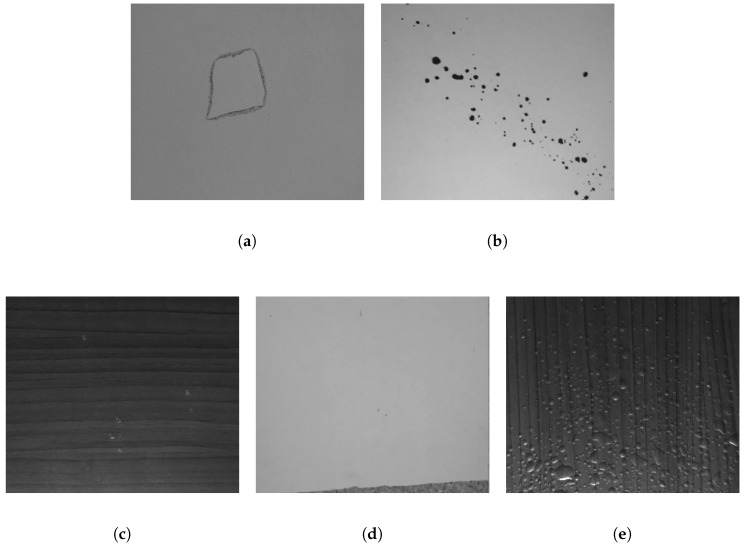
Example of different defects that can appear in melamine boards ((**a**)Paper scrap, (**b**) Stains, (**c**) White spots, (**d**) Paper displacement, (**e**) Bubbles).

As stated in the previous section, at each step of the fabrication process a defect could appear that will alter the final quality of the product. In this work, we focus on classifying five different types of defects, paper scrap, stains, white stops, paper displacement, and bubbles (see Figure 1). Each one of those defects appears due to one or more of the following reasons, the presence of dust or suspension particles, dirt or zones without adhesives.

The classification of the previously described defects is key to the commercialization of the product in demanding markets. Nowadays these detection and classification problems are manually performed, or with equipment that just uses VS cameras. Thus in this work, we test following hypotheses: The usage of images from different spectrum could improve the classification performance of defects, in contrast with the sole use of images from the visible spectrum.

## 3. Proposed Approach

A SVM [3] was used in this study to classify the defects in wood-based panels. SVM is a widely used method, that can learn from small quantity of data, in contrast with more advanced techniques that require much more data, which make it suitable for our case study. As input of the classifier we use a feature based approach, testing E-LBP features and SURF using a Bag of Words [20] representation.

The E-LBP method proposed in Liu et al. in 2012 [19] was adopted, which is a generalization of [21], the original method. From the many variants of LBP have been proposed [22], E-LBP still produces high performance in texture descriptors benchmarks, even when comparing with more novel convolutional neural networks (CNN) based texture descriptor approaches such as [23,24] that are more sensitive to image degradations [25].

In E-LBP, two types of operators are presented: intensity and differences. The intensity-based operators consider the intensity of the central pixel (CI) and its neighborhood (NI), while those based on differences are based on the radial difference (RD) and the angular difference (AD). For each pixel and for the operators NI, RD and AD, the “invariant uniform patterns to rotation” (riu2) are calculated. These new operators are a compressed form of those initially calculated and are characterized by joining all the “non-uniform” patterns of the image into a single pattern, leaving only the patterns that may have some uniformity in their local texture. Considering all the pixels of the image, riu2 histograms of these 3 operators are generated, obtaining NI-LBPriu2, RD-LBPriu2 and AD-LBPriu2. These concatenated histograms, together with the CI histogram, correspond to the descriptor for a specific radius value. To address the problem of scale in the image patterns, the method includes a multi-resolution scheme, calculating descriptors for different resolutions, that is, for different radius values. In this way, the final E-LBP descriptor that represents the entire image under analysis will be the concatenation of the descriptors calculated for the different radius values.

The BoW model is a type of image representation and is currently one of the most recognized methods in the characterization of objects, scenes or textures. Basically, it consists in the clustering of a set of interest points or keypoints of an image in different “words” defined in the BoW. The result is represented by a histogram of these “words”, where the length of this histogram is given by the number of words of the BoW already defined. This bag of visual words is also called dictionary or visual vocabulary. The extraction of the BoW from an image can be summarized in the following steps [20]:

1. Obtain interest points of the image. This step is commonly carried out in the interest point detection stage of some local description algorithm. In the present work, the SURF descriptor [12] was used, which provides the keypoints from which the descriptors are calculated.
2. Calculate the descriptors corresponding to the obtained keypoints. The SURF algorithm provides descriptors of 64 elements, corresponding to the descriptor of each keypoints of the image.
3. Generate a of vocabulary or visual dictionary. To generate this dictionary, a set of training images is used, from which steps 1 and 2 are executed. From the total of calculated descriptors, the k-means clustering algorithm is applied, where k is the number of words in the dictionary, resulting in k groups of descriptors.
4. Find the occurrences of each “word” of the dictionary in the set of SURF descriptors of each image. In this way we obtain the BoW of an image, which is an histogram of length k, which represents the occurrence of the SURF descriptors in each word of the dictionary generated in the training stage.

## 4. Experimental Setup

This section details the multispectral camera rig used to acquire the set of images that are evaluated in the experiments. Additionally, it describes the calibration and rectification methods together with the steps followed to heat each panel sample. Panels are heated to emulate real industrial conditions during the manufacturing process.

### 4.1. Multispectral Camera Rig

**Figure 2:**
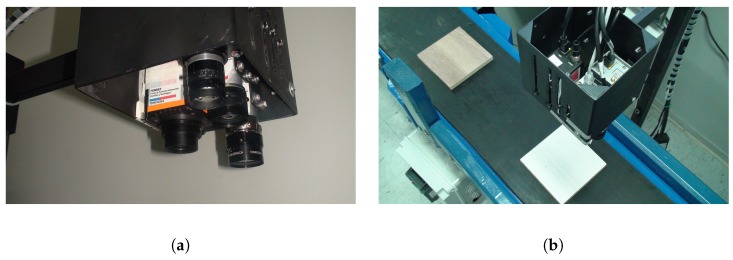
Experimental setup ((**a**) Multispectral camera rig, (**b**) Conveyor prototype).

The multispectral camera rig consists of four different cameras placed in a two-row configuration as depicted in Figure 2a. Two cameras work in the infrared spectrum, one on the LWIR wavelength and the other on the NIR wavelength. Regarding the other two cameras, one works on the visible spectrum and the other on the UV spectrum. Although our multispectral camera rig includes an UV camera, after preliminary studies we decided not to use it during the experiments, since the UV camera under UV light did not provide any relevant information concerning the defects. An halogen light source that covers the VIS and NIR spectral bands was used during the acquisition of the image samples.

The VIS camera (BASLER acA645-100gm) captures images with a resolution of 659 × 494 pixels, the NIR camera (BASLER acA2000-50gm) captures images with a resolution of 2048 × 1080 pixels and the LWIR camera (Xenics Gobi-640-GigE) captures images with a resolution of 640 × 480 pixels. The three cameras are connected to a computer through Ethernet connection, using a switch (LINKYS LGS108P).

### 4.2. Calibration and Rectification

Images of each camera were calibrated and rectified using the Matlab calibration toolbox from [26]. The calibration and rectification processes aim to reduce the effects of the distortion that the optics of each camera can generate, obtaining non-distorted images that later on are manually aligned.

**Figure 3:**
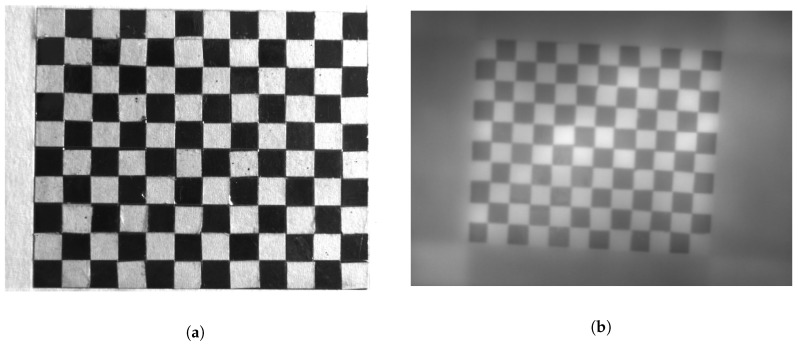
Calibration Pattern ((**a**) Calibration pattern in the VIS spectrum, (**b**) Calibration pattern in the LWIR spectrum).

A key element used to calibrate the cameras is the calibration pattern, which is a rigid metal plate with alternating black and white squares aligned like a chessboard, that is used as fixed positions references for the calibration algorithm. Figure 3 shows two images of the calibration pattern, one taken with the VIS camera and the other with the LWIR camera, prior to the calibration process.

### 4.3. Heating Process

Melamine boards come out from the press in the manufacturing process at high temperature, prior to the quality control stage. To emulate this condition, each sample is heated using a griddle to a temperature of approximately 140${}^{{}^{\circ}}$ for ten seconds—different heating times have been empirically tested (5 to 30 s), obtaining the best thermal contrast after 10 s of heating. After each sample is heated, the images are immediately captured with each camera simultaneously.

### 4.4. Dataset

An essential part of this work was the acquisition of a multispectral melamine faced panels dataset, that contained the defects described in Section 2 together with free of defects parts. Table 1 contains the details of the number of boards that were used to generate the dataset along with the number of defects that where acquired for each category, including the free-of-defects parts. In average, the size of board samples were of 15×15 cm${}^{2}$.

The procedure to extract each defect sample for a board is as follow.

1. A sample is manually aligned between all the spectral bands.
2. Manually, a region of interest is defined over each defect present in the board by an expert.
3. Randomly, regions outside the regions of interest are selected to be free-of-defect samples.
4. Randomly, some samples are selected to be training samples and other testing samples.

**Figure 4:**
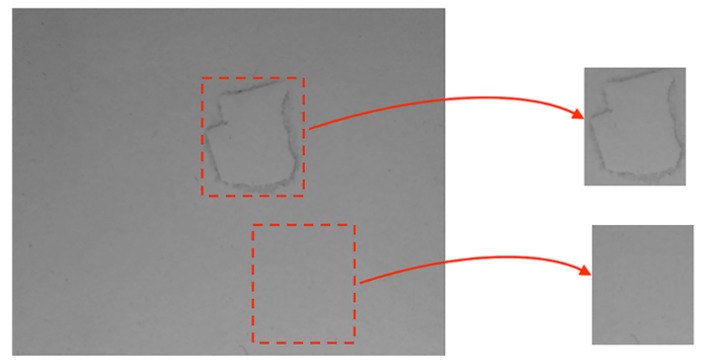
Regions of interest generation for a defect and a non-defect area, in a melamine faced board.

Figure 4 shows examples of defects and non-defects areas selected by the procedure mentioned above.

## 5. Experimental Results

In order to evaluate the usage of images from different spectral bands to classify defects in melamine faced panels, a multiclass classification approach (one vs all) was implemented, using the dataset described in Section 4.4. In particular, three experiments were carried out during this work, one using only images from one spectral band at the time, another experiment using a naive early fusion scheme—i.e., combining images as the average sum—and finally one experiment using a late fusion scheme—i.e., appending the descriptors of the different images.

**Figure 5:**
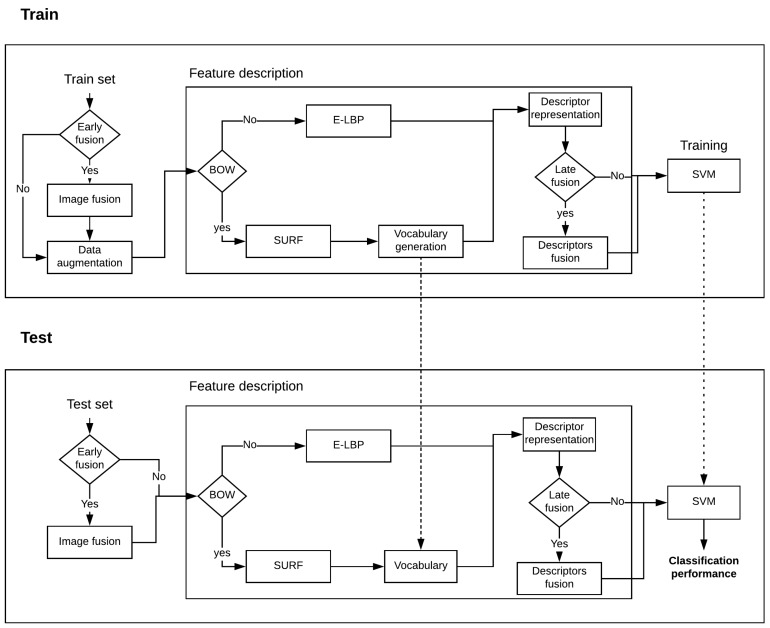
Block diagram of training and testing phases.

For each combination of approaches and spectral bands, a parameter search was performed using a grid search and cross-validation with k = 5. We used an RBF kernel for the SVM, searching for two parameters C (from 1 to 10) and sigma (1 to 10). All the implementation was carried out using Matlab. Additionally, data augmentation techniques were used to increase the size of the training data by ten folds during the training process, where the augmented data was obtained by rotating the images in four angles, scaling the images in three scales, adding noise, and translating the images in four different directions. Figure 5 shows a block diagram of training and testing phases.

### 5.1. Single Band

**Figure 6:**
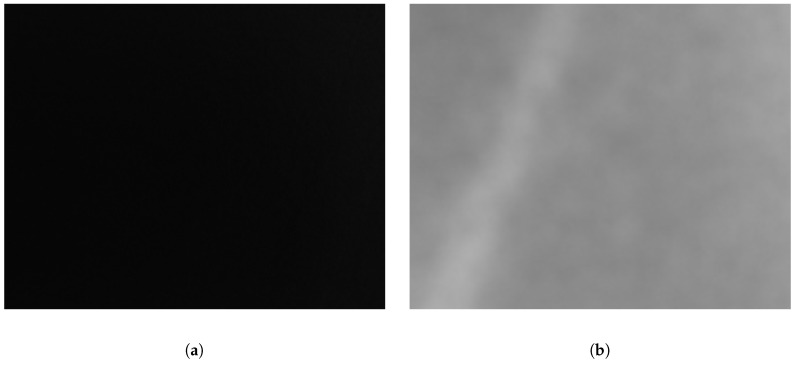
Example of a wood defect in a dark color melamine faced panel. In the visible spectrum the defect cannot be appreciated, while in the LWIR spectrum it can be easily detected (the defect is the white rectangle with a slope at the left of the image) ((**a**) VS spectrum, (**b**) LWIR spectrum).

Single band experimental results are shown in Table 2. Results show that the classification performance is closely related between the combination of the descriptor and spectral band. For example, E-LBP performed better than other solutions, but in average BoW was better. More importantly, both non-visible solutions performed better than the VS one. Additionally, some detection of defects are clearly improved by the use of non-visible cameras, like the paper scraps. Moreover, our dataset contained a small number of dark melamine boards, that are hard to classify with VS images that could have increased the difference in performance with non-visible images (see Figure 6).

### 5.2. Early Fusion

In general, early fusion results (Table 3) are not much better than the single band solution. However, the best overall performance in all our experiments was with the combination of the NIR and VS spectral bands using BoW. Definitively, more complex fusion scheme will benefit the performance of the solution.

### 5.3. Late Fusion

Late fusion results (Table 4) were similar to early fusion results. However, even with the combination of different spectral bands, was not possible to overpass the single band solution results. This means that an early fusion scheme is more useful than a late one for this specific classification problem.

## 6. Conclusions and Future Work

Through experimental evaluation, this work shows that using infrared images for the classification of defects in melamine faced panels can achieve better classification performance than visible-based solutions. Moreover, our best results were obtained when a combination of NIR and VS images were considered in a naive early fusion scheme.

Although our results show the benefits of using images beyond the visible spectrum in the classification of melamine faced panels, more data are needed to have more conclusive results. Additionally, more complex fusion schemes must be explored that can take full advantage of the different spectral bands used in the experiments.

References

## Figures and Tables

**Table 1:** Defect type and number of samples analyzed.

Type	Boards	Training Samples	Testing Samples
Paper scraps	16	9	32
Staints	37	25	25
White spots	27	25	25
Paper displacement	20	10	40
Bubbles	36	22	88
Without defect	136	90	89

**Table 2:** Classification performance using E-LBP/BoW and single spectral band (PS = Paper scraps, ST = Stains, WS = White spots, PD = Paper displacements, and BB = Bubbles, WD = Without defect).

Feature	Spectral Band	PS	ST	WS	PD	BB	WD	Total Accuracy
E-LBP	NIR	**1.00**	**0.84**	**1.00**	0.97	**0.89**	**0.95**	**0.94**
	VS	0.75	0.76	**1.00**	**1.00**	0.54	0.94	0.80
	LWIR	0.75	0.24	0.84	**1.00**	0.40	0.82	0.66
BoW	NIR	**0.96**	0.88	0.92	0.87	0.87	**1.00**	0.92
	VS	0.87	**0.96**	**1.00**	0.80	**0.88**	**1.00**	0.92
	LWIR	**0.96**	0.92	**1.00**	**0.95**	0.81	**1.00**	**0.93**

**Table 3:** Classification performance using E-LBP/BoW and naive early fusion (PS = Paper scraps, ST = Stains, WS = White spots, PD = Paper displacements, and BB = Bubbles, WD = Without defect).

Feature	Fusion	PS	ST	WS	PD	BB	WD	Total Accuracy
E-LBP	VS-LWIR	**1.00**	0.760	0.92	**1.00**	0.61	**0.97**	0.85
	NIR-LWIR	0.84	**0.84**	**1.00**	**1.00**	0.69	0.96	0.87
	NIR-VS	0.75	0.76	0.96	0.85	0.84	0.94	0.86
	NIR-VS-LWIR	0.84	0.76	**1.00**	0.87	**0.86**	**0.97**	**0.90**
BoW	VS-LWIR	0.93	0.72	0.96	**0.95**	0.73	**1.00**	0.88
	NIR-LWIR	0.93	0.76	0.88	0.77	0.71	**1.00**	0.84
	NIR-VS	0.90	**0.92**	**1.00**	0.90	**0.96**	**1.00**	**0.96**
	NIR-VS-LWIR	**0.96**	0.76	**1.00**	0.82	0.92	**1.00**	0.93

**Table 4:** Classification performance using E-LBP/BOW and late fusion (PS = Paper scraps, ST = Stains, WS = White spots, PD = Paper displacements, and BB = Bubbles, WD = Without defect).

Feature	Fusion	PS	ST	WS	PD	BB	WD	Total Accuracy
E-LBP	VS-LWIR	0.75	0.76	**0.92**	**1.00**	0.71	**0.97**	0.85
	NIR-LWIR	**0.87**	**0.84**	**0.92**	0.95	0.85	0.96	0.90
	NIR-VS	**0.87**	0.76	0.96	0.97	**0.87**	0.95	**0.91**
	NIR-VS-LWIR	0.62	0.76	0.88	**1.00**	0.72	0.96	0.839
BoW	VS-LWIR	**1.00**	0.84	**1.00**	0.87	0.78	**1.00**	0.90
	NIR-LWIR	0.96	0.80	0.92	0.87	0.79	**1.00**	0.89
	NIR-VS	0.93	**0.92**	**1.00**	0.87	**0.89**	**1.00**	**0.94**
	NIR-VS-LWIR	**1.00**	0.88	0.92	**0.92**	0.81	**1.00**	0.92
